# Rabbit antiserum to mouse embryonic stem cells delays compaction of mouse preimplantation embryos

**Published:** 2014-01

**Authors:** Yingli Cong, Lifang Cui, Zhenhong Zhang, Jianzhong Xi, Mianjuan Wang

**Affiliations:** 1 *College of Pastoral Agriculture Science and Technology, Lanzhou University, Lanzhou, Gansu 730020, China.*; 2 *General Grassland Station of Xinjiang, Urumqi, Xinjiang 830049, China.*; 3 *College of Animal Science and Technology, Agricultural University of Hebei, Baoding, Hebei 071001, China.*; 4 *School Hospital of Agricultural University of Hebei, Baoding, Hebei 071001, China.*

**Keywords:** *Mouse*, *Embryonic stem cells*, *Immune sera*, *Preimplantation development*, *Chimera*

## Abstract

**Background:** Mouse embryonic stem (ES) cells are derived from the inner cell mass (ICM) of the preimplantation blastocysts. So it is suggested that ES and ICM cells should have similar cellular surface molecules and antiserum to ES cells can inhibit ICM development.

**Objective: **The objective of this study was to evaluate the effect of rabbit antiserum to ES cells on mouse preimplantation embryo development and chimera production.

**Materials and Methods:** Mouse 4-cell embryos were matured in vitro at 37.5^o^C, in humidified 5% CO_2_ atmosphere for 12-36 h. The embryos were cultured in KSOM medium with or without antiserum for 12-36 h. The ratios of in vitro embryo development of the blastocysts, cell division, attachment potential, alkaline phosphatase activity, post-implantation development, and chimera production were assessed and compared with the control group. P<0.05 was considered as significant.

**Results:** The rabbit antiserum to mouse ES cells showed delay in embryo compaction and induced decompaction at 8-cell stage. The development of 4-cell embryos in the presence of the antiserum for 36h did not lead to a reduced or absent ICM. These embryos still displayed positive alkaline phosphatase activity, normal cell division, embryo attachment, outgrowth formation, implantation and post-implantation development. In addition, decompaction induced by antiserum did not increase production and germline transmission of chimeric mice.

**Conclusion:** The results showed that antiserum to ES cells delayed embryo compaction and did not affect post-implantation development and chimera production.

## Introduction

Mouse embryonic stem (ES) cells, derived from the inner cell mass (ICM) of the preimplantation blastocysts, are undifferentiated, immortal cells capable of differentiating into derivatives of all three embryonic germ layers and show a deficiency in extra embryonic lineages (1). ES cells may have similar cellular surface molecules with ICM cells rather than trophectoderm (TE) cells. So we suggested that anti-mouse ES cells antibody can inhibit ICM formation during blastocyst development. 

A previous document demonstrated that a rabbit antiserum to a mouse embryonal carcinoma cell (EC) line blocks compaction of cleaving mouse embryos. Cell division is not affected up to the 32-cell stage but intracellular junctions fail to develop. Removal of the antibody at this stage permits compaction to occur and a normal blastocyst develops (2). Compared with EC cells, ES cells have more identical cellular surface molecules to ICM. We suggested that antiserum to ES cells have more effective inhibition roles on ICM formation than antiserum to EC cells. In addition, monoclonal antibody to E-cadherin blocks compaction of cleaving mouse embryos and leads to a reduced or absent ICM (3, 4). 

Also polyclonal antiserum to ES cells is contained antibody to E-cadherin (5-7). Based on these documents described above and our deduction, it was strongly hypothesized that antiserum to ES cells can inhibit ICM formation during blastocyst development. Fully ES-cells derived mice will be produced when mouse ES cells are injected into this kind of ICM-free blastocyst if the hypothesis is true. The method is similar to tetraploid embryo complementation assay (8, 9). 

Also we hypothesized even if ICM can’t be completely blocked by the anti-mouse ES cell antibody; the limited developmental blastocysts may be more suitable for the production of chimeric mice. When ES cells are injected into this kind of blastocysts with limited developmental ICM, they have more chances to contribute to derivatives of all three embryonic germ layers.

## Materials and methods

**Mice**

In this experimental study, CD-1 mice were purchased from Beijing Vitalriver Laboratory Animal Technology (Beijing, China). The mice were housed at 25^o^C under 50-60% relative humidity with a 12h light: 12h dark photoperiod (lights on at 06:00 AM) until they were used in this experimental study. Mice were fed with commercial pelleted food and water *ad libitum*. All experimental protocols and animal handling procedures were reviewed and approved by the Laboratory Animal Care and Use Committee of Agricultural University of Hebei, China.

**Antiserum**

For the establishment of hybrid ES cells, blastocysts were collected 3.5 days postcoitum from C57BL/6 females mated to DBA/2 males (Vital River, Beijing, China). Blastocysts were cultured in ES cell media on a feeder layer; at day 5 the outgrowth was dissociated by pipetting in trypsin solution, and the cell suspension was replated on a fresh feeder layer. These plates were screened 4 days later for the presence of ES cell colonies. 

ES cell media was Dulbecco’s modiﬁed Eagle’s medium (DMEM; Gibco, Shanghai, China) with 20% fetal calf serum (Gibco, China) containing 1,000 units/ml of leukemia inhibitory factor (Gibco, China) on mitomycin C-treated mouse embryonic ﬁbroblasts, as previously described (10). The rabbit antiserum to mouse ES cells was prepared as previous described (11). 

Briefly the feeder cells were removed by feeder-free culture and harvested by 0.02% EDTA solution. The cell suspension was washed extensively in phosphate-buffered saline (PBS) and 10^8^ cells in PBS were emulsified in Freund's complete adjuvant (FCA) [1 volume antigen: 2 volumes FCA] and injected into multiple subcutaneous sites of a New Zealand White rabbit in 2-3 weeks intervals. After three injections the animal was boosted with 10^7^ cells intravenously and bled 10 days later. The separated serum was heat inactivated by incubation for 30 min at 56^o^C and stored in small aliquots.

**Recovery and culture of embryos**

4-5 weeks old female mice, were super-ovulated with 10 units of serum gonadotropin (Tianjin Laboratory Animal Center, China) from pregnant mares and then with 10 units of human chorionic gonadotropin (HCG), 48 hr later to induce ovulation. After the HCG injection, the females were mated with CD-1 males. The presence of a vaginal plug in the next morning was taken as evidence of mating and gestation. After 54-56h post-HCG, the females were killed by cervical dislocation and the oviducts were removed and flushed to recover 4-cell embryos. All embryo culture was carried out in micro-drops on standard bacterial petri dishes (Nunc, China) under mineral oil (Sigma, China). 

KSOM media (potassium simplex optimized medium) with or without antiserum was used for embryo culture. M_2_ media was used for room temperature operations whereas long-term culture was carried out in bicarbonate-buffered KSOM at 37.5^o^C with an atmosphere of 5% CO_2_ in air. We first confirmed that exposure to antiserum resulted in embryo decompaction and delayed blastocyst formation. Early 4-cell embryos were placed either in control medium (with or without rabbit serum) or experimental medium containing antiserum, respectively, and then cultured to the early blastocyst stage.

**Cell counting**

Embryos were cultured in KSOM media containing 5 µg/ml Hoechst 33342 (Sigma, China) for twenty minutes, and then were pressed on slides and the cell number of each embryo was counted under an inverted fluorescence microscope. 

**Embryo** **postimplantation development** **and alkaline phosphatase staining**

To investigate the roles of antiserum on embryo postimplantation development, the control and treatment embryos were seeded on mouse embryonic fibroblast (MEF) feeders or transferred into uterine horns of 2.5 days postcoitum pseudo pregnant CD-1 females. After antiserum culture for 36h, embryos were seeded on MEF feeders inactivated with 10 µg/ml Mitomycin C (Sigma, China) in 35-mm gelatin-coated tissue culture dishes (Nunc, China) to view ICM outgrowth formation. 

The culture medium consisted of Dulbecco’s modified Eagle’s medium (DMEM; Invitrogen) supplemented with 20% (v/v) bovine serum (Invitrogen), 2 mM glutamine, 0.1 mM nonessential amino acids (Invitrogen), 0.1 mM 2-mercaptoethanol, 50 IU/ml penicillin and 50µg/ml streptomycin (Invitrogen) under standard conditions (37^o^C, 5% CO_2_, saturated humidity). The ICM outgrowth was characterized by morphology and alkaline phosphatase staining. Histochemical staining for alkaline phosphatase was carried out using a commercially available kit, according to the manufacturer’s instructions (Chemicon, China).

**Embryo microinjection and transfer**

It was suggested that ES cells injected into decompacted embryos may have more opportunity and advantage to locate in centre of embryos and form ICM and then contribute to derivatives of all three embryonic germ layers. To address this hypothesis, decompacted embryos were injected with 10 mouse ES cells according to previous described (11) and cultured overnight in KSOM media including antiserum and then transferred into each uterine horn of 2.5 days postcoitum pseudo pregnant CD-1 females that had mated with vasectomized males. Pregnant recipients were subject to a Caesarean section on day 10.5 of pregnancy or natural delivery. Pups from Caesarean section were placed upon a warming stage and respiration was observed. Live fetuses were counted and fostered to lactating CD-1 mothers. Adult chimeric mice were mated with CD-1 females to verify their germline transmission by coat color.

**Statistical analysis**

Statistical evaluation was performed using chi-square. Mean±SD was reported for descriptive analysis. SPSS software version 16 was used for statistical analysis. Statistical significant was set at p<0.05. For cell counting, one-way ANOVA was used to determine if there was significant variance amongst these data, which, if found, was then followed by the Tukey-Kramer multiple comparisons test to perform pairwise comparisons between individual means (InStat, GraphPad Software, San Diego), and the level of significance was set at p<0.05.

## Results

**Antiserum delayed embryo compaction and blastocyst formation**

The antiserum was cleared of any precipitate and diluted 1/50, 1/100, 1/200 in medium KSOM, and further tested on the embryos in preliminary experiments. A dilution of 1/100 was found to give consistent and complete decompaction without any deleterious or toxic effects on the embryos. Lesser or greater dilutions of antiserum produced more variable decompaction. To compare it with control embryos in KSOM with or without a dilution of 1/50 rabbit serum, cultures of 4-cell embryos in 1/50 dilution of antiserum significantly inhibited cleavage and caused some blastomeric death, and 1/200 antiserum produced weak decompaction. Thereafter a dilution of 1/100 antiserum in medium KSOM was used in this experiment.

**Figure 1:**
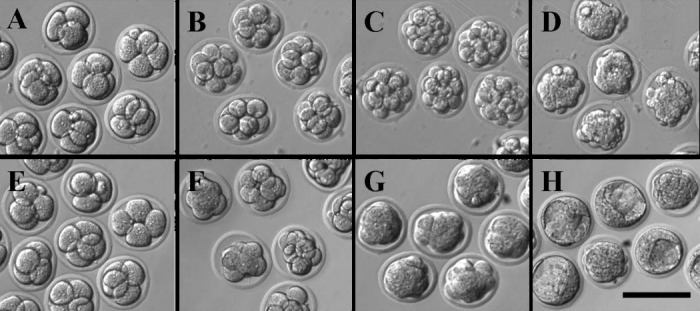
Antiserum delayed embryo compaction and blastocyst formation.

On 12h culture, there were no significant differences among these groups. On 24h culture, 160 out of 170 embryos exposed to antiserum suffered decompaction while 116 of 134 embryos in medium KSOM and 144 out of 156 embryos in medium KSOM with rabbit serum developed to compacted morula stage. On 36h culture, 94.1% of embryos exposed to antiserum developed to compacted morula stage while 80.8-82.3% of control embryos in medium KSOM with or without rabbit serum have developed to early blastocyst stage (Table I, Figure 1).

**Antiserum did not affect division of embryonic cells**

Continuous incubation of 4-cell embryos in antiserum did not appear to affect division of cells up to the blastocyst stage. After 36h culture, the number of embryonic cells in KSOM, KSOM+ rabbit serum, and KSOM+ rabbit antiserum was 35.2±8.5 (n=45), 33.5±10.7 (n=40), and 33.8±11.1 (n=37), respectively. There were no significant differences among groups. 

**Antiserum did not affect ICM outgrowth and embryo postimplantation development**

**Figure 2:**
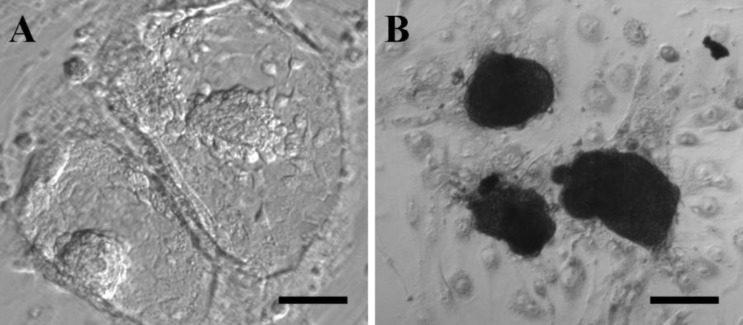
Antiserum did not affect ICM outgrowth formation.

**Figure 3:**
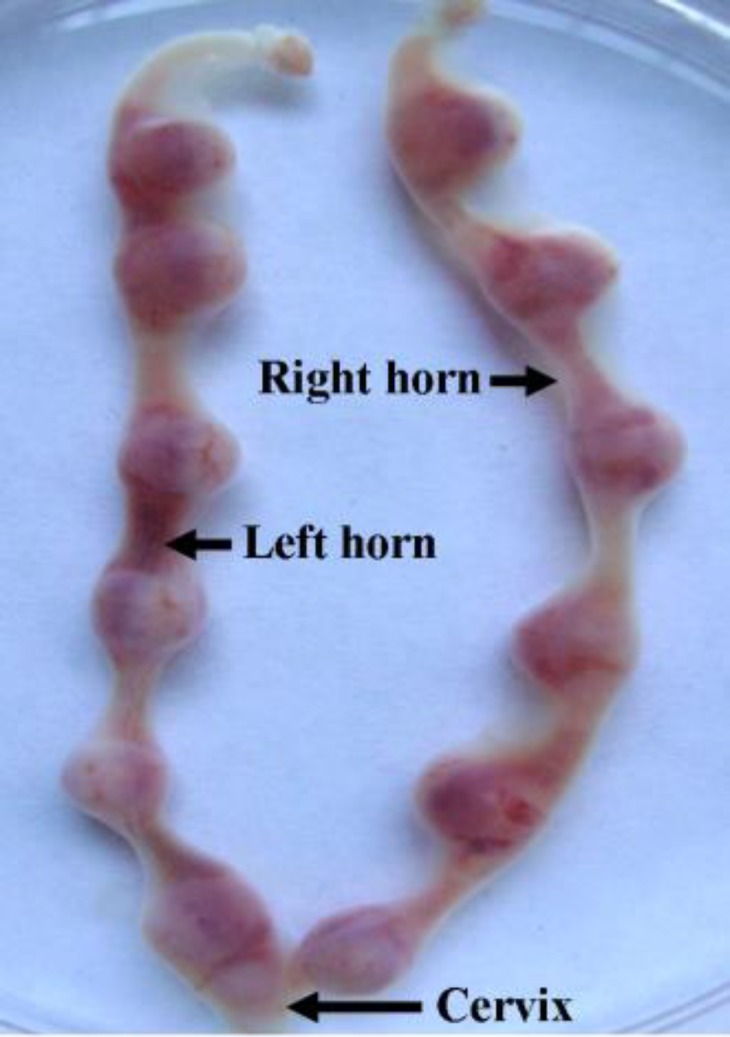
Antiserum did not affect embryo postimplantation development.

Almost all of embryos seeded on MEF feeder produced ICM outgrowths (Table II) and there were no significant differences among different groups after even after 3-day culture. All of ICM outgrowths were positive of alkaline phosphatase staining (Figure 2). When embryos were transferred into uterine horns and viewed on E10.5, all of the embryos normally implanted and developed, and no morphological differences were observed (Figure 3). 

**Decompaction-induced by antiserum did not affect chimera production**

**Figure 4:**
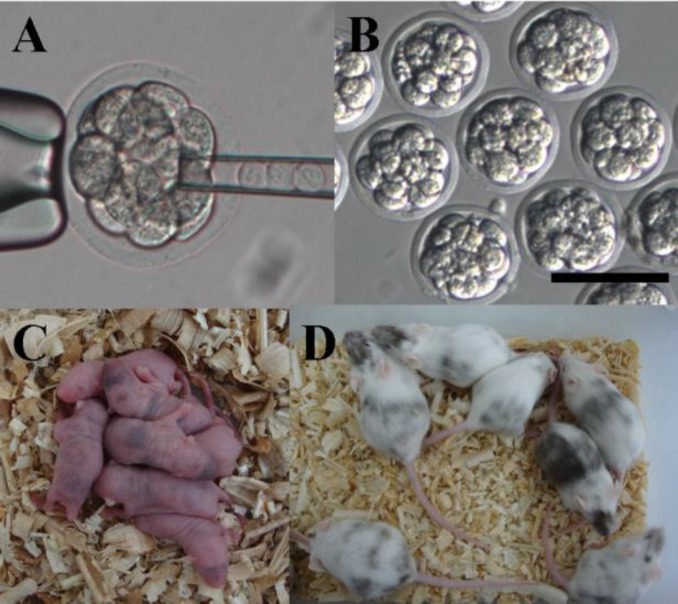
Decompaction embryos induced by antiserum were injected with mouse ES cells and then produced chimeric mice.

Rabbit antiserum induced embryo decompaction and delayed embryo compaction. The role of antiserum may be favorable to production of chimeric mice. In this study, 152 decompacted embryos were injected with 10 mouse ES cells and cultured overnight in KSOM media including antiserum and then transferred into uterine horn of 2.5 days postcoitum pseudo pregnant CD-1 females. 18 coat chimeric mice were generated and 15 were germline transmissions. As a control, 180 morulae were injected, cultured in KSOM media without antiserum and transferred into 2.5 days pseudo pregnant mice. 25 coat chimeric mice were generated and 21 were germline transmissions (Figure 4). Our data showed that decompaction did not increase production and germline transmission of chimeric mice.

**Table I:** Rabbit antiserum delays mouse embryo compaction and blastocyst formation

**Media/Groups**	**4-cell embryos**	**8-cell embryos (%)/ Compacted embryos (%)** *	**Decompacted embryos (%)/ Compacted embryos (%) ** **	**Compacted embryos (%)/ Blastocysts (%) ** ***
KSOM	134	104 (83.9%)/ 16 (12.9%)	0 (0)/ 116 (93.5%) ^a^	14 (11.3%)/ 102 (82.3%)^ a^
KSOM with serum	156	140 (89.7%)/ 10 (6.4%)	0/ 144 (92.3%)^ a^	18 (11.5%)/ 126 (80.8%)^ a^
KSOM with antiserum	170	164 (96.5%)/ 0 (0)	160 (94.1%)/ 0 (0)^ b^	160 (94.1%) /0 (0)^ b^

Note: Different letters in the same row means significant difference between the treatments by chi-square test (p<0.05).

* after 12 h culture

** after 24h culture

*** after 36h culture

**Table II:** Roles of antiserum on ICM outgrowth *in vitro* and implantation *in vivo*

**Media/Groups**	**Embryos seeded on MEF feeder**	**Outgrowths**	**Embryos transferred into uteri**	**Implantation sites**	**Newborns**
KSOM	70	62 ^a^	52 (64)	40^ a ^(-)	- (38^a^)
KSOM with serum	84	72 ^a^	60 (72)	46^ a^ (-)	- (44^a^)
KSOM with antiserum	92	78^ a^	82 (74)	60^ a ^(-)	- (40^a^)

Note: The same letters in the same row means no significant difference between the treatments by chi-square test (p<0.05).

Decompaction embryos were injected with 10 mouse ES cells (A, B) and were cultured overnight and then transferred into uterine horns of 2.5 days postcoitum pseudopregnant CD-1 females. C, newborn chimeric mice; D, adult chimeric mice. The scale bar is 100µm.

## Discussion

The results described here demonstrate that rabbit antiserum to mouse ES cells blocks embryo compaction and induces decompaction at 8-cell stage. The development of embryos in the presence of the antiserum did not lead to a reduced or absent ICM and embryo postimplantation development. The 4-cell embryos cultured in antiserum showed positive alkaline phosphatase activity, normal cell division, embryo attachment, outgrowth formation, implantation and fetus development. In addition, decompaction induced by antiserum did not increase production and germline transmission of chimeric mice. It was hypothesized that mouse ES cells have more identical cell surface molecules with ICM rather than TE cells because ES cells were derived from ICM. Compared to monoclonal antibodies to a specific cell surface molecule, polyclonal antiserum to ES cells can effectively inhibit ICM development and produce ICM-free blastocysts. 

In this study, rabbit antiserum to mouse ES cells blocked embryo compaction but did not inhibit ICM and postimplantation development. This indicates that compaction is not a prerequisite for blastocyst formation or the emergence of ICM and TE: the first distinct cellular differentiation event during early embryo development. However previous similar documents demonstrated that both monoclonal antibody to E-cadherin and polyclonal antiserum to a mouse EC cell line blocks compaction of cleaving mouse embryos and leads to a reduced or absent ICM (2-5). 

This indicates that ES cells and EC cells have identical cell surface antigens. But how can antiserum to mouse EC cells inhibit ICM and antiserum to mouse ES cells cannot? This is a so interesting fact and suggested that EC cells have more identical surface antigens to ICM than ES cells. At the same time, the fact hinted a subtle relationship between ES cells and EC cells. This was beneficial for us to decide whether ES cells are abnormally differentiated EC cells or EC cells are abnormally differentiated ES cells. In addition, the role of decompaction of antiserum can be removed or reduced if antiserum was absorbed with ES cells or mouse embryonic fibroblasts, respectively (data not shown). It is known that antiserum to ES cells or EC cells was undoubtedly directed against many factors, and it was not therefore possible to ascribe the blocked compaction to inactivation of any specific cell surface molecule (2, 12). Unlike other agents who block compaction, the two kinds of antiserum did not appear to affect the rate of cell division up to the 32-cell stage at which time control embryos are forming blastocysts (2).

Our results showed that antiserum inhibit compaction at the 8-cell stage but not inhibit compaction at early blastocyst stage. Decompacted embryos by antiserum at the 8-cell stage were compacted again at 32-cell stage even in the continuous presence of antiserum. This indicates that the embryos become resistant to antiserum during development, suggesting that cell-cell adhesion systems, such as tight junctions, which cannot be blocked by antiserum, develop in cells in the surface layer of embryos at a later stage. 

Therefore, the compaction of embryos occurring at the 8-cell stage and around the 32-cell stage should involve different processes. This was consistent with a previous report in which E-cadherin antibody induces two independent phases in compaction (3, 13). In preliminary experiments we observed that the antiserum has the same decompaction as monoclonal antibody to E-cadherin. The decompaction will lost when the antiserum were neutralized with E-cadherin (data not shown). *Zona pellucida* was not removed in this study. This probably blocked some antibodies to go inside of embryos and partially decreased the effects of antiserum. Further investigation will be necessary to detect antiserum using *zona*-free embryos at different stages in the next step.

A current hypothesis proposes that the polarization of blastomeres, dependent on cell contact and compaction, is an essential step for generation of cells directed to ICM lineage (5, 14). However in this study, decompaction induced by antiserum to ES cells did not impair ICM formation and postimplantation development. The results to differ from the previous documents where decompaction induced by E-cadherin antibody or antiserum to EC cells, impair ICM cells (2-5). It was necessary to further investigate if our antiserum affects polarization of blastomeres. In addition, our cell counting data indicated that rabbit anti-mouse serum did not reduce cell number in ICM by Hoechest staining. However, cell number of the whole blastocyst might cover the difference of ICM between the experimental and control groups. Cell number of ICM and TE will be examined respectively in the next step.

When ES cells were injected into decompacted embryos, the cells have plenty of time to locate in the centre of embryos and have more chances to form ICM cells. So we suggested that antiserum treatment can increase production and germline transmission of chimeric mice. But we did not find the effects in this study. This indicates that decompaction or delayed compaction does not increase chimera production.
